# Deep feature loss to denoise OCT images using deep neural networks

**DOI:** 10.1117/1.JBO.26.4.046003

**Published:** 2021-04-23

**Authors:** Maryam Mehdizadeh, Cara MacNish, Di Xiao, David Alonso-Caneiro, Jason Kugelman, Mohammed Bennamoun

**Affiliations:** a The Australian e-Health Research Centre, CSIRO, Western Australia, Australia; b University of Western Australia, School of Physics, Mathematics and Computing, Western Australia, Australia; c Queensland University of Technology, School of Optometry and Vision Science, Contact Lens and Visual Optics Laboratory, Centre for Vision and Eye Research, Queensland, Australia

**Keywords:** optical coherence tomography, convolutional neural networks, image enhancement, speckle, image processing

## Abstract

**Significance:** Speckle noise is an inherent limitation of optical coherence tomography (OCT) images that makes clinical interpretation challenging. The recent emergence of deep learning could offer a reliable method to reduce noise in OCT images.

**Aim:** We sought to investigate the use of deep features (VGG) to limit the effect of blurriness and increase perceptual sharpness and to evaluate its impact on the performance of OCT image denoising (DnCNN).

**Approach:** Fifty-one macula-centered OCT pairs were used in training of the network. Another set of 20 OCT pair was used for testing. The DnCNN model was cascaded with a VGG network that acted as a perceptual loss function instead of the traditional losses of $L_{1}$ and $L_{2}$. The VGG network remains fixed during the training process. We focused on the individual layers of the VGG-16 network to decipher the contribution of each distinctive layer as a loss function to produce denoised OCT images that were perceptually sharp and that preserved the faint features (retinal layer boundaries) essential for interpretation. The peak signal-to-noise ratio (PSNR), edge-preserving index, and no-reference image sharpness/blurriness [perceptual sharpness index (PSI), just noticeable blur (JNB), and spectral and spatial sharpness measure (S3)] metrics were used to compare deep feature losses with the traditional losses.

**Results:** The deep feature loss produced images with high perceptual sharpness measures at the cost of less smoothness (PSNR) in OCT images. The deep feature loss outperformed the traditional losses ($L_{1}$ and $L_{2}$) for all of the evaluation metrics except for PSNR. The PSI, S3, and JNB estimates of deep feature loss performance were 0.31, 0.30, and 16.53, respectively. For L1 and L2 losses performance, the PSI, $S_{3}$, and JNB were 0.21 and 0.21, 0.17 and 0.16, and 14.46 and 14.34, respectively.

**Conclusions:** We demonstrate the potential of deep feature loss in denoising OCT images. Our preliminary findings suggest research directions for further investigation.

## 1 Introduction

Optical coherence tomography (OCT) is an imaging modality that allows for the noninvasive assessment and identification of the internal structures of the retina. The OCT image quality can often be degraded by speckle noise, which is inherent to this imaging technique. The properties of OCT backscattering signal and the associated speckle behavior have been studied in detail in Refs. 1 and 2. From a visual inspection perspective, the presence of speckle noise obscures subtle but important morphological details and thus is detrimental in clinical diagnosis. Speckle noise also negatively affects the automatic analysis methods intended for objective and accurate quantification of the images.

The goal of denoising methods is to reduce the grainy appearance in homogeneous areas, while preserving the image content, particularly boundaries that represent the transition between retinal layers. These retinal layer boundaries are the most commonly used clinical information to extract thickness data^3^ and make subsequent clinical decisions. These data are commonly extracted using automatic segmentation methods.

According to the number of frames collected in the same retinal location during a single acquisition, OCT image denoising methods that are available in the literature can be categorized under single frame denoising^4^^–^^11^ and multiple-frame denoising.^12^^–^^14^ Among the single frame approaches, block matching and 3D filtering^11^ and complex wavelet-based K-SVD^10^ methods have demonstrated promising performance; however, they can introduce artifacts by operating in the wavelet domain. Among the multiple-frame denoising methods, multiscale sparsity-based tomographic denoising^13^ has shown a superior performance compared with single-frame and other multiple-frame approaches.

While these methods have demonstrated an improved performance, they provide inadequate noise reduction under high levels of speckle noise, resulting in a significant loss of subtle image features.^10^^,^^11^^,^^13^ In addition, these methods require the careful selection of numerous parameters of the learning algorithm, which is also not adaptive to various levels of noise. The development of more advanced denoising methods to provide minimum loss of details, while also minimizing the requirement for handpicked parameters, is a challenging task of OCT image denoising.

In recent years, deep learning methods, including convolutional neural networks (CNNs), have been applied to OCT image denoising; these include DeSpecNet,^15^ cGAN,^16^ SDSR-OCT,^17^ and perceptually sensitive OCT denoising.^18^ These deep learning methods show good performance; however, it is worth noting that the results for the proposed deep learning methods are generally reported using the peak signal-to-noise ratio (PSNR) metric as well as visual inspection of the OCT images. Other metrics such as edge preservation or contrast are not always reported in these studies, despite their importance to obtaining a comprehensive understanding of the method’s performance.

To date, research has focused on the definition of new architectures that can be applied to OCT image denoising. Most of the proposed architectures use loss functions that aim to minimize the pixelwise differences between the “predicted” output image and the ground truth “averaged” OCT image. Although high PSNRs are reported, which is indicative of good performance, using pixelwise loss for training can result in aggressive denoising, or smoothing, which can compromise important features such as crispness of retinal layer boundaries and textures/features that are critical for medical diagnostics. It is reasonable to assume that OCT images reside on a nonlinear image manifold, where the pixelwise similarity does not reflect the true intrinsic similarity between images but just their “brute-force” Euclidean distances. For example, two identical images shifted by only one pixel may be very different as measured by pixelwise distances, despite being perceptually similar. Therefore, some features that are critical for diagnosis might be lost during the denoising process.

Neural networks progressively compare the predicted output of the network with the “ground truth” using a loss function, which is an effective driver of the network’s learning. For instance, Qiu et al.^18^ proposed a method that utilized SSIM and MSE in their denoising network as perceptual losses. They demonstrated that their approach can outperform other related denoising methods to preserve the structural details of the retinal layers and improve the perceptual metrics. In contrast, Zhang et al.^19^ showed that the classic per-pixel measures that are commonly used for regression problems, such as the Euclidean distance, are not suitable for assessing the perceptual similarity between images. For example, they showed that blurring causes large perceptual but small $L_{2}$ changes. They also revealed that PSNR and SSIM are simple, shallow functions and fail to account for many complexities of human perception. They demonstrated that the internal activations of deep networks, trained for high-level classification tasks, correspond better to human perceptual judgments. The authors introduced the learned perceptual image patch similarity (LPIPS) metric as a similarity measure, demonstrating that LPIPS provides a closer distance between the original image patch and a sharp but distorted patch than between the original and a more similar (under $L_{2}$) but blurry patch.

Several earlier studies^20^^–^^27^ employed the features extracted from pretrained deep networks for various computer vision tasks such as style transfer^21^ and deep feature visualization.^25^^–^^27^ It was observed that the features extracted from trained deep networks are descriptive of the contents of images. This culminated in the paper by Zhang et al.,^19^ who defined a metric to reflect the contextual similarity between images and to investigate how that similarity is aligned with the perceptual similarity between images. The common message from this collective research is that features extracted from pretrained deep convolutional networks, even across architectures (Squeezenet,^28^ AlexNet,^29^ and VGG^30^) provide an emergent embedding, which agrees remarkably well with the complexities of human perception of image similarities, much better than the widely used traditional perceptual metrics such as PSNR and SSIM. However, the ability for deep features to act as a “perceptual loss” to drive the training of an OCT denoising network and how this compares with the traditional losses such as $L_{2}$ and $L_{1}$ are yet to be explored. Employing deep features as an advanced perceptual metric, compared with traditional perceptual metrics such as PSNR and SSIM, sets our work apart from the perceptually sensitive OCT denoising work by Qiu et al.^18^

To summarize the contributions of this paper, we first bring attention to the importance of the loss function used to train DnCNN for OCT image denoising. Despite the well-known limitations of pixelwise losses such as $L_{2}$ and $L_{1}$, these losses are still widely used in training feedforward networks such as DnCNN. Second, we investigate the use of deep VGG features as loss functions for training the DnCNN network as well as the contribution of individual convolutional layers in the VGG pretrained network in addition to the combination of layers. We hypothesize that not all levels of feature abstraction are equally useful for OCT denoising. Looking at the whole VGG16 network and giving equal weights to all layers is conceptually just “averaging out” each layer’s performance into a single conglomerate. Third, we perform our experiments on OCT images to demonstrate the effectiveness of deep feature loss in comparison with traditional pixelwise losses. In addition, in this study, we emphasized the importance of considering metrics beyond the commonly used PSNR to capture the complete behavior of the denoising network.

## 2 Methodology

In this paper, we evaluate the use of deep features learned through the VGG network as a loss function to train DnCNN,^31^ a well-known denoising network, for the purpose of OCT image denoising. To achieve this, the DnCNN model is cascaded with a VGG network that acts as a perceptual loss function. The VGG network is pretrained on ImageNet and remains fixed during the training process.

We view the OCT denoising problem as a classical image transformation task in which inputs are mapped from a noisy OCT image space to the averaged OCT image space. In our approach, DnCNN provides a mapping function from noisy to denoised image spaces, while the VGG-based deep feature loss guides the learning process so that image content is best preserved during the transformation.

### 2.1 Noise Reduction Model

Let $x\in R^{N\times N}$ denote a noisy OCT image and $y\in R^{N\times N}$ denote the corresponding averaged “speckle-free” OCT image. The goal of the denoising network is to learn a transformation T that maps the noisy image x to the averaged image y:

T:x↦y. (1)

During the training phase, the network updated itself so that, after the transformation T, the denoised image (output prediction) was as close as possible to the averaged OCT image (ground truth, speckle-free image).

Speckle noise in OCT images represents a physical phenomenon that is complex to model. Therefore, it is complicated to learn a mapping function from the noisy OCT image space to the averaged OCT image space. However, deep learning networks have been shown to be effective at learning such a complicated transformation function with a modest number of training images.

### 2.2 DnCNN

The input of the DnCNN network was a noisy image. DnCNN network adopted the residual learning formulation to train a residual mapping R(x)≈v, where v is the noise and the residual formulation is y=x−v. The DnCNN network was developed to model noise from noisy images. The optimization problem was formed such that the difference between the noisy input x and the noise v is as close as possible to the clean image y.

**Fig. 1:**
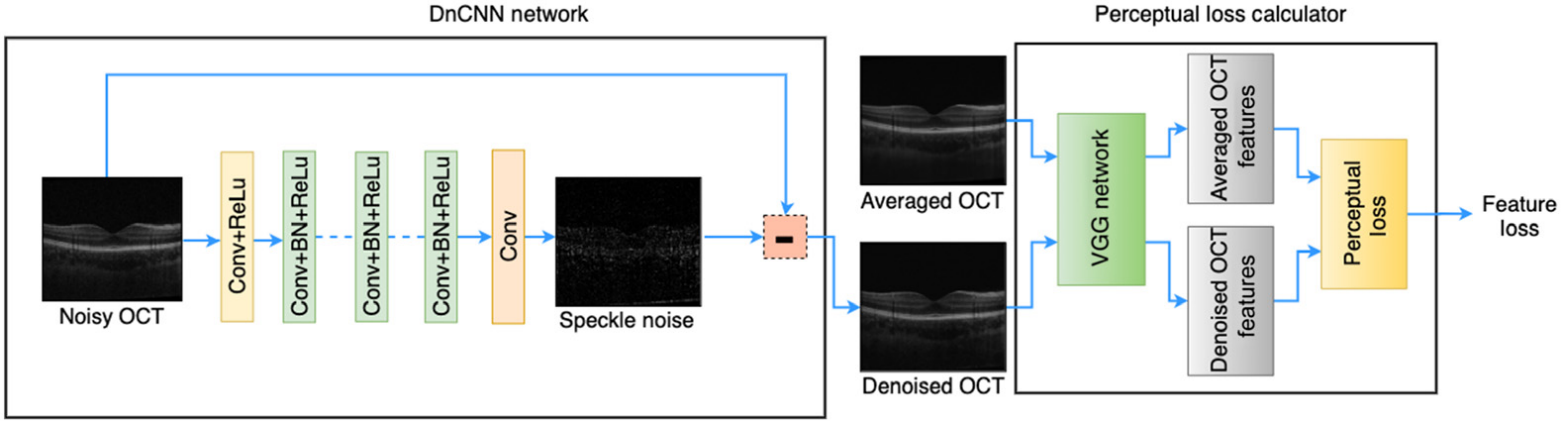
The overview of the cascaded DnCNN-VGG network.

The optimization problem is formulated as minimizing loss l, where

$$l(Q)=\frac{1}{n}\overset{n}{\underset{i=1}{\sum }}{\left\|R(x_{i},Q)-(y_{i}-x_{i})\right\|}^{2},$$ (2)

where n is the batch size and Q are trainable parameters in the DnCNN network. In the original implementation of this work,^31^ the $L_{2}$ norm is used. The loss function therefore reflects the averaged Euclidean distance between the clean images and the predicted outputs. The DnCNN network consists of 17 convolutional layers of three different types as shown in Fig. 1: (i) Conv+relu for the input layer, (ii) Conv+BN+relu for the second layer to the penultimate layer, and (iii) Conv for the output layer. For all layers except the output layer, 64 kernels of size 3×3 are used. To ensure that the spatial dimensions of the inputs and outputs are the same, in all layers a stride of 1 and “‘same” padding are used. For the output layer, 1 kernel of size 3×3 is used.

### 2.3 Deep Feature Loss

The original DnCNN network is designed to use the Euclidean pixelwise loss. Therefore, while the DnCNN network is learning a transformation function to map the image from a noisy space to a denoised image space, the loss function calculates the Euclidean distance between the output patches and gold standard image patch.^15^

Two of the most common losses used in feedforward denoising deep networks are $L_{1}$ and $L_{2}$ pixelwise losses.

$L_{2}$ loss is the sum of the squared difference of pixels of two image patches $y_{i}$ and $x_{i}$:

$$L_{2}(T)=\frac{1}{n}\overset{n}{\underset{i=1}{\sum }}{\left\|T(x_{i})-y_{i}\right\|}_{2}^{2}.$$ (3)

$L_{1}$ loss is the sum of the absolute difference of pixels of two image patches $y_{i}$ and $x_{i}$:

$$L_{1}(T)=\frac{1}{n}\overset{n}{\underset{i=1}{\sum }}{\left\|T(x_{i})-y_{i}\right\|}_{1}^{1}.$$ (4)

The perceptual loss is the sum of the squared differences of features extracted from the pretrained network:

$$L_{\text{Perceptual}}(T)=\frac{1}{n}\frac{1}{whd}\overset{n}{\underset{i=1}{\sum }}{\left\|\phi (T(x_{i}))-\phi (y_{i})\right\|}^{2},$$ (5)

where ϕ is the pretrained deep network, n is the batch size, and w, h, and d are the width, height, and depth of the convolutional layers, respectively.

In our experiments, we employed the VGG-16 network pretrained on ImageNet^32^ as the deep feature loss calculator:

$$L_{\mathrm{VGG}}(T)=\frac{1}{n}\frac{1}{whd}\overset{n}{\underset{i=1}{\sum }}{\left\|\mathrm{VGG}(T(x_{i}))-\mathrm{VGG}(y_{i})\right\|}^{2}.$$ (6)

From now on, we refer to the features extracted by the VGG-16 network as the VGG loss.

**Fig. 2:**
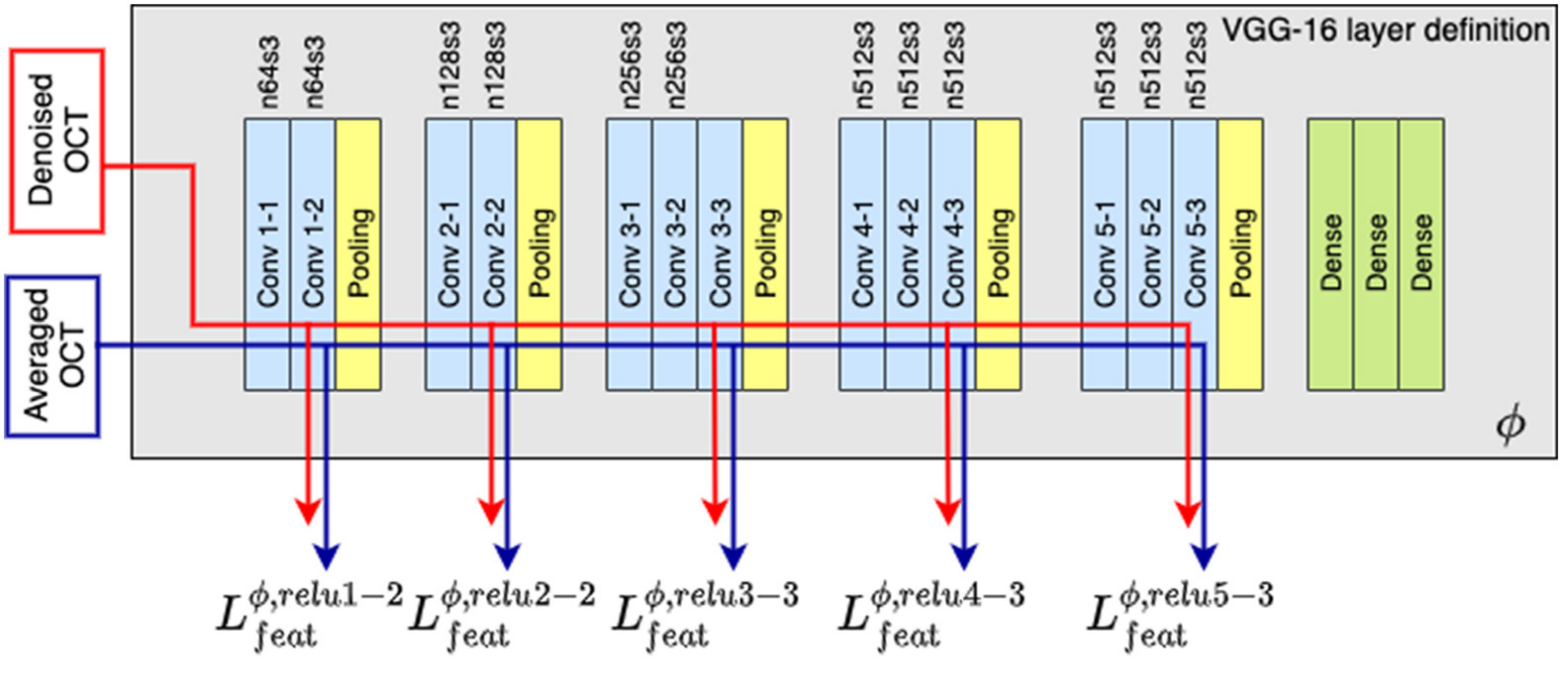
VGG-16 network used to extract the loss function; this network consists of five main convolutional blocks. The feature loss is calculated from the feature outputs of relu1-2, relu2-2, relu3-3, relu4-3, and relu5-3. “n” represents number of filters, and “s” represents strides.

The weights of the VGG-16 network were kept unchanged during the training of the DnCNN network (Fig. 2). The predicted outputs of the DnCNN network were grayscale images. VGG-16 network expects a three channel (RGB) input, so here the input is a gray-scale OCT image replicated three times. The VGG-16 network contains 13 convolutional layers and 3 dense layers. Motivated by the approach in Ref. 20, the output of the 2nd, 4th, 7th, 10th, and 13th convolutional layers are used as the extracted features from the VGG-16 network.

### 2.4 Network Architecture

Figure 1 displays an overall view of the cascaded networks of DnCNN and VGG, which we call DnCNN-VGG. The transformation network (DnCNN) is a feedforward CNN. The last layer generates one feature map with a single 3×3 filter, which was subtracted from the input image to generate the final output of the denoising DnCNN network.

The denoising network was followed by the deep feature loss calculator. Figure 2 shows the architectural details of the VGG-16 network consisting of five convolutional layers with 64, 128, 256, and 512 filters, respectively. The predicted outputs of the DnCNN network and their corresponding averaged OCT image were then passed to the VGG-16 network for feature extraction. The Euclidean distance between the extracted features of VGG-16 layer(s) formed the objective loss of the network as indicated by Eq. (6). The deep feature loss is then backpropagated to the DnCNN network to update the trainable parameters.

The relu-VGG reconstruction loss is computed from all individual layer feature losses, as follows:

$$\mathrm{relu}-\mathrm{VGG}=\lambda_{1}(\mathrm{relu}1-2)+\lambda_{2}(\mathrm{relu}2-2)+\lambda_{3}(\mathrm{relu}3-3)+\lambda_{4}(\mathrm{relu}4-3)+\lambda_{5}(\mathrm{relu}5-3),$$ (7)

where the $\lambda_{1}$, $\lambda_{2}$, $\lambda_{3}$, $\lambda_{4}$, and $\lambda_{5}$ are the scalar weighting factors.

**Fig. 3:**
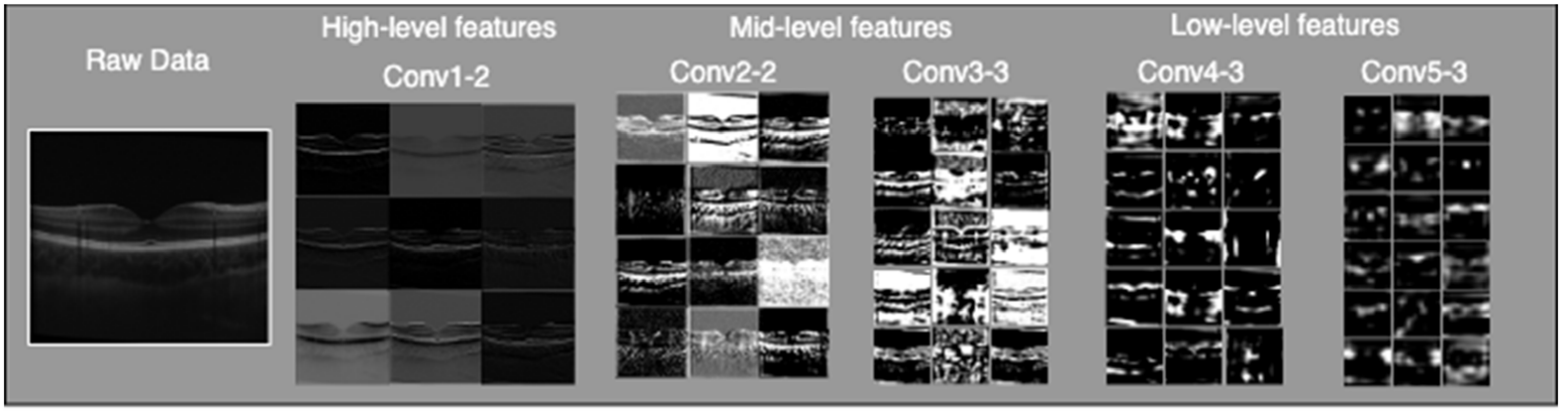
Visualization of the feature maps extracted from the high, middle, and lower convolutional layers in the VGG16 model. It can be appreciated that the initial layers provide high-level features closer to the input of the model, while the deeper layers in the VGG model show more abstract features (low level) of the input image.

We investigate the contribution of each layer in extracting features from the OCT images, and how these features support the network with the denoising task. Figure 3 provides a visualization of the feature maps output by the VGG16 network when an OCT image is input to the network. As we go deeper through the VGG16 network, the number of feature maps increases, while the size of the feature maps decreases. The high level feature maps capture a lot of fine details in the image. The deeper layer feature maps contain abstract features that are suitable to perform classification; however, we generally lose the ability to visually interpret the deeper feature maps. In this study, we will establish what level of feature abstraction from the VGG16 network (pixelwise can be considered the zeroth level of abstraction) provides the “best” metric for OCT noise reduction with the least amount of blurring and the highest edge preservation. We design VGG loss networks to be a subset of the layers in the full VGG16 model. The model has the same input layer as the original VGG16 model, but the output would be the output of a certain convolutional layer. As we go deeper in the VGG16 layers, the number of feature maps (depth or channels) increases, while the size of the feature maps decreases.

We experimented with two sets of weights for the VGG network.

• Pretrained VGG-16 network weights were trained on ImageNet.^32^
• Pretrained VGG-16 network weights were further calibrated on a large-scale database of perceptual judgements. The weights and dataset were introduced by Ref. 20 and can be publically accessed. For the rest of this paper, we refer to them as LPIPS.

**Fig. 4:**
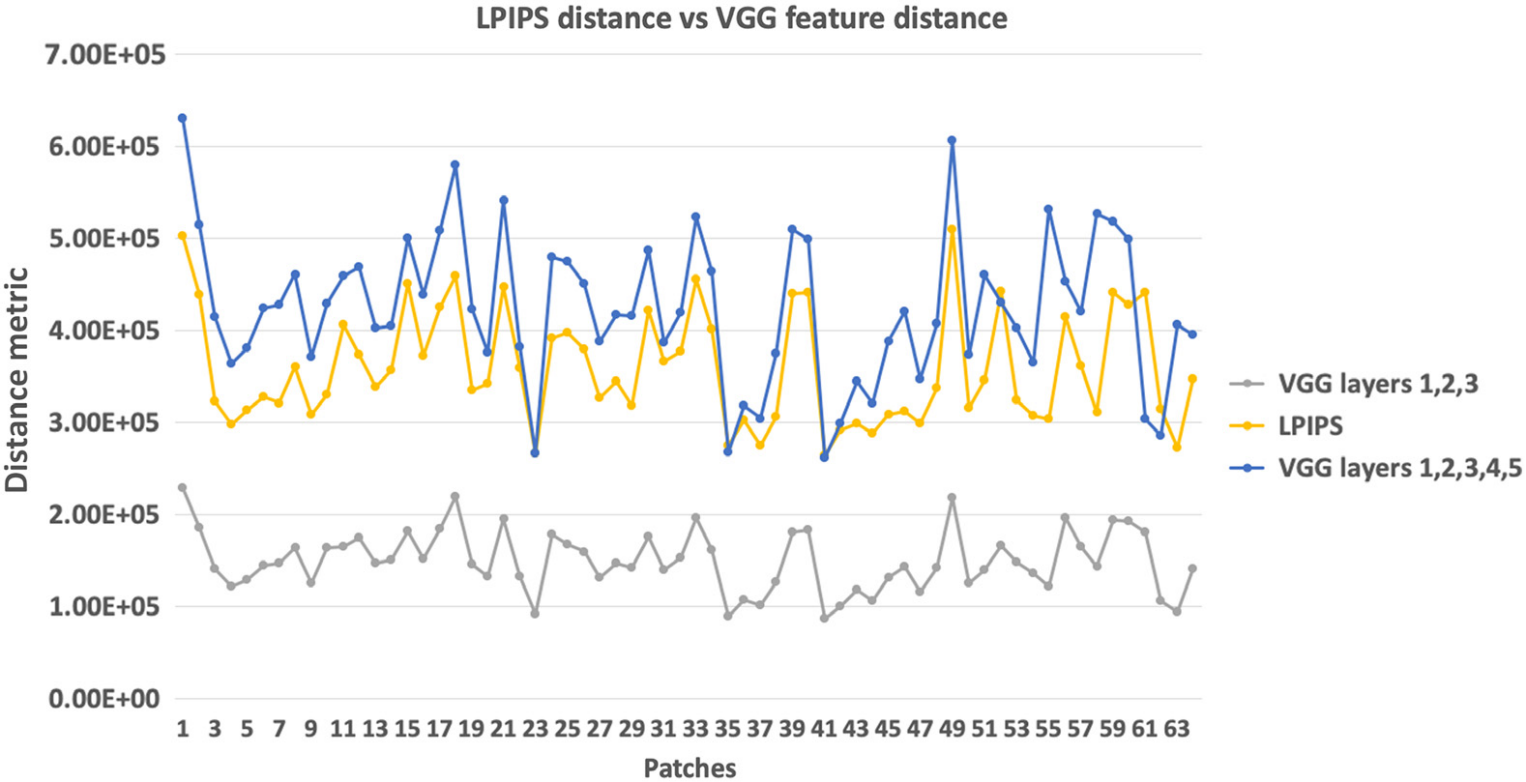
Comparison of distance metric calculated by LPIPS versus VGG. The graph illustrates the LPIPS, the VGG distance calculated from all layers (1 to 5) and the VGG distance calculated from first three layers (1 to 3). The layers are given equal weights while calculating the feature distance. The distances were calculated between 64 OCT patch pairs of noisy and averaged images.

A comparison of the feature distances for a set of 64 OCT image noisy and averaged pairs is displayed in Fig. 4. It can be seen that the LPIPS and VGG resemble the same trend for “perceptual similarity” of OCT image patches.

### 2.5 Trained Networks

For comparison purposes, we trained the following networks:

• DnCNN-$L_{2}$ with $L_{2}$ loss;
• DnCNN-$L_{1}$ with $L_{1}$ loss;
• DnCNN-VGG with VGG feature loss;
• DnCNN-VGG with individual layer VGG feature loss;
• DnCNN-$L_{1}\text{-}L_{2}$-VGG combination of pixelwise losses and VGG loss;
• DnCNN-LPIPS with LPIPS image patch similarity loss.

## 3 Experiments and Results

### 3.1 Experimental Dataset

**Fig. 5:**
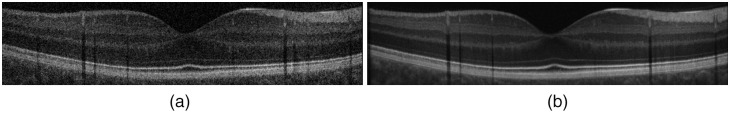
(a) Original OCT B-scan image and (b) the corresponding averaged OCT B-scan.

We perform our experiments on a dataset that was originally introduced by Ref. 33. The data comprise foveal centered OCT retinal scans of 226 children aged between 4 and 12 years with normal vision in both eyes and no history of ocular pathology. The images were acquired using a spectral-domain OCT instrument (Copernicus SOCT-HR Optopol Technology SA, Zawiercie, Poland). The dataset consists of OCT noisy scans at the same retinal location and the corresponding averaged “noise-free” OCT image pairs (Fig. 5)—each measuring 999×868  pixels—along with eight different retinal layer boundaries. The averaged image is acquired by registering and averaging several B-scans obtained at the “same” retinal position.^34^

In our experiments, 51 OCT image pairs were randomly selected (noise and corresponding averaged) to train the DnCNN network. Similarly, a separate set of 20 OCT image pairs was randomly selected for testing and validation. We divided the images into overlapping 180×180  pixels with a stride of 45 pixels. No further data augmentation was performed on the data. Patches that did not contain any retinal structures were removed from the analysis.

### 3.2 Network Training

In our experiments, the Adam optimizer^35^ was used across all networks with a learning rate α=0.001 and a mini-batch size of 128. During testing, no cropping was applied to the images; the whole B-scan was input to the trained network. The deep learning framework was implemented on Tensorflow, and all experiments were conducted on four GPU nodes on the Pawsey supercomputing facility, each node having 2x Intel Xeon Broadwell E5-2680 v4, 14-core CPUs (28 cores total) @ 2.4 GHz (nominal), 256 GB of RAM, and 4x NVIDIA Tesla P100s (each card has 16 GB memory). Each network training (over 100 epochs) took about 5 h. Each network was trained five times, and the model with the highest PSNR was chosen for each network.

**Fig. 6:**
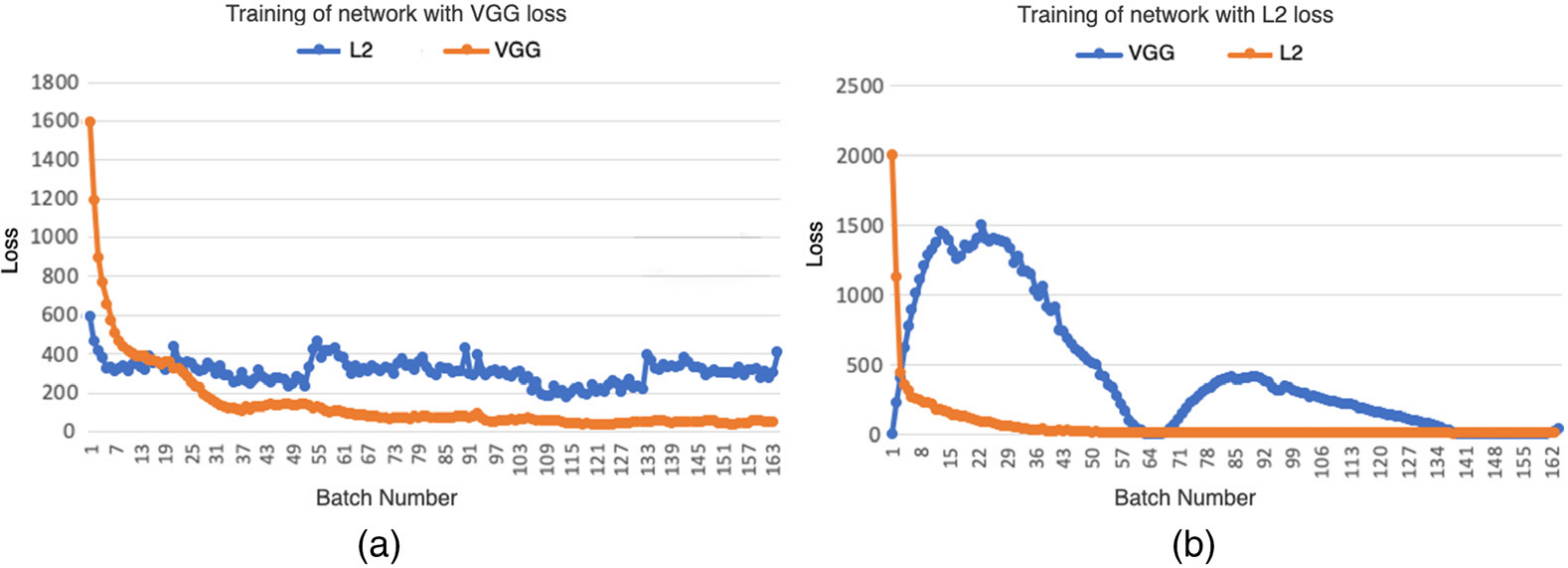
(a) With training the network with VGG loss, the $L_{2}$ loss and VGG loss are correlated. (b) With training the network with pixelwise loss, the $L_{2}$ loss and VGG loss are not correlated.

To visualize the convergence of the networks, we calculated the VGG loss and $L_{2}$ loss according to Eqs. (3) and (6) over the 8903 image patches that were used for validation. Since the overall trend for the losses was from high to low, a small representative window of loss (over 164 steps in the first epoch) is presented to demonstrate the difference in trend of the two losses. For a network trained with the VGG loss, the VGG loss and $L_{2}$ loss decreased smoothly together, which suggests that a VGG loss produces results that are correlated with the $L_{2}$ loss results. On the other hand, for the network trained with the pixelwise loss, the $L_{2}$ loss decreases, while the VGG loss increases. This suggests that the $L_{2}$ loss is optimizing the network with a different focus than the VGG loss. The difference between pixelwise losses and deep feature losses will be further discussed in the next section. Results for these initial training steps are shown in Fig. 6.

### 3.3 Qualitative Analysis

**Fig. 7:**
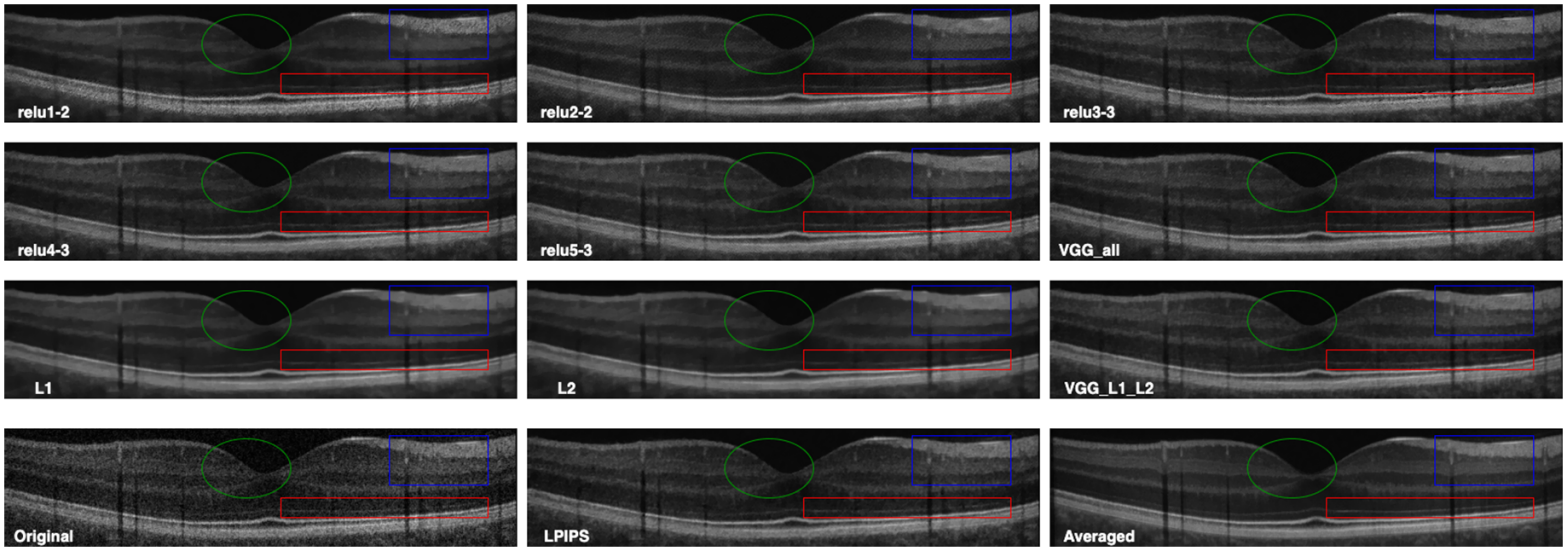
First row from left to right are the denoised images from the DnCNN-Conv1-2 (relu1-2), DnCNN-Conv2-2 (relu2-2), and DnCNN-Conv3-3 (relu3-3) networks. The second row shows the denoised images from the DnCNN-Conv4-3 (relu4-3), DnCNN-Conv5-3 (relu5-3), and DnCNN-VGG (VGG-All) networks. The third row shows the denoised images from the DnCNN-$L_{1}$ ($L_{1}$), DnCNN-$L_{2}$ ($L_{2}$), and DnCNN-VGG-$L_{1}\text{-}L_{2}$ (VGG-$L_{1}\text{-}L_{2}$) networks. The fourth row shows the original noisy OCT image, the denoised OCT by DnCNN-LPIPS (LPIPS), and the averaged OCT image.

**Fig. 8:**
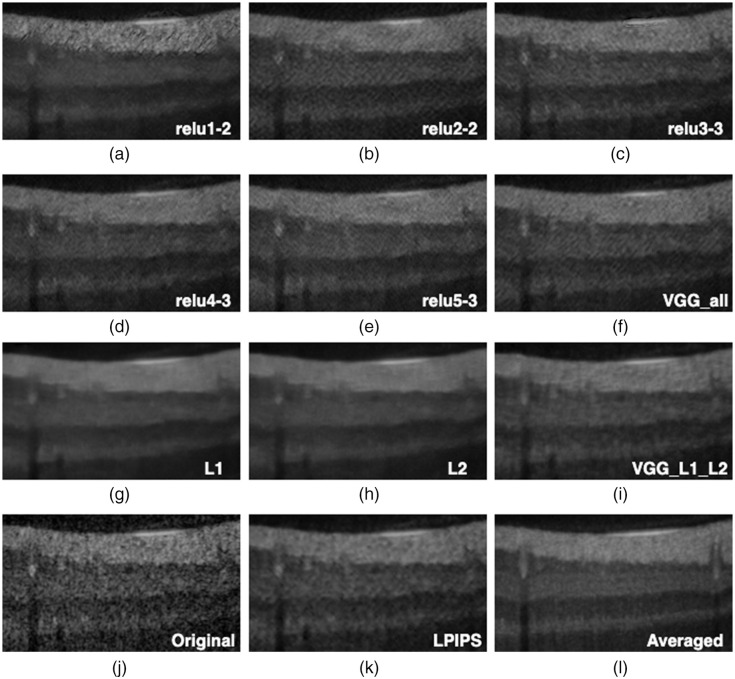
This ROI corresponds to the highlighted blue rectangle in Fig. 7. These pictures show the contrast between retinal layers and the blood vessels.

**Fig. 9:**
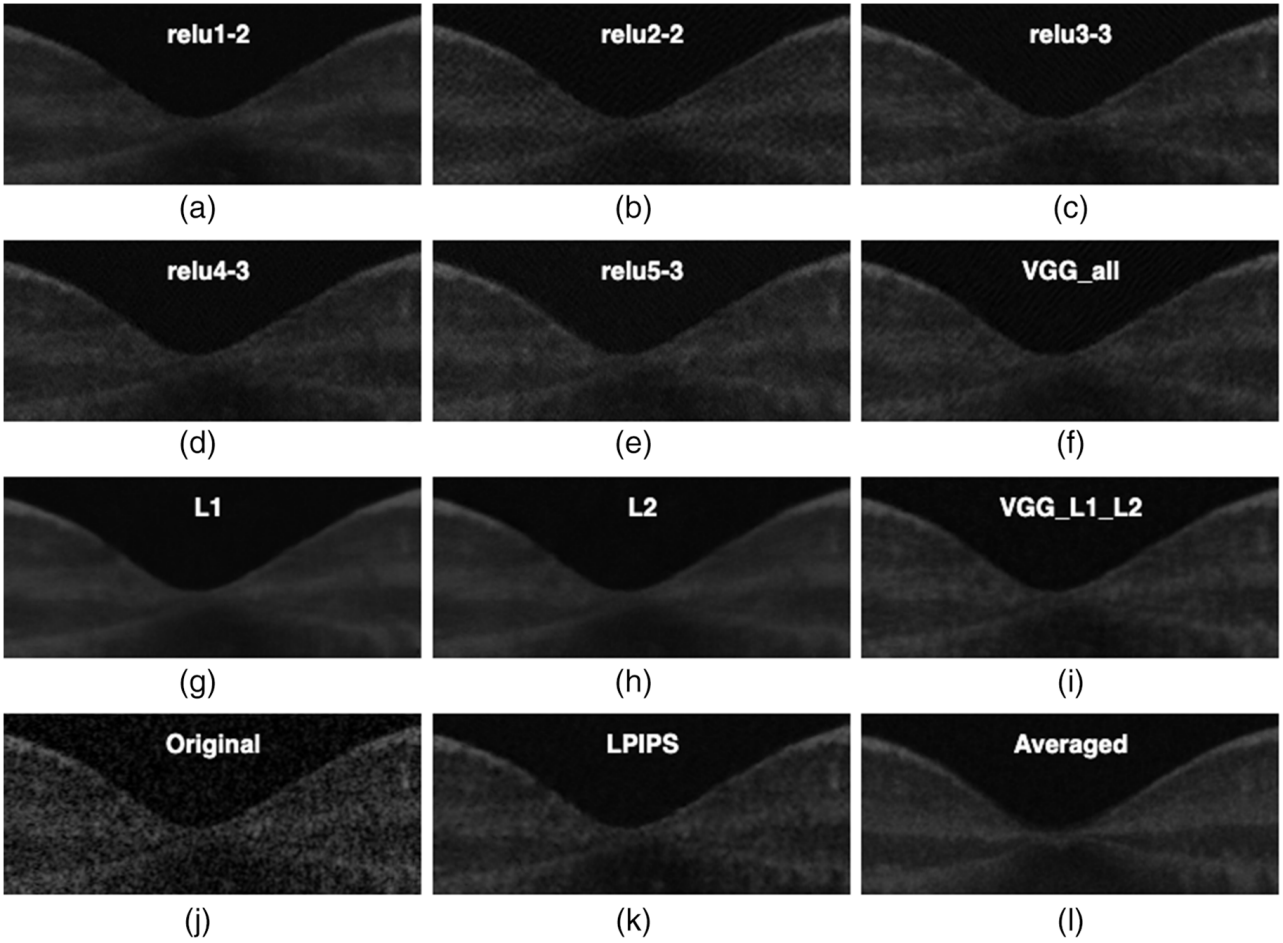
This ROI corresponds to the highlighted green oval shape in Fig. 7. These pictures magnify the macular region, the contrast between the background, and the retinal layers. The contrast between retinal layers is most faded by the DnCNN-$L_{1}$ and DnCNN-$L_{2}$ networks.

**Fig. 10:**
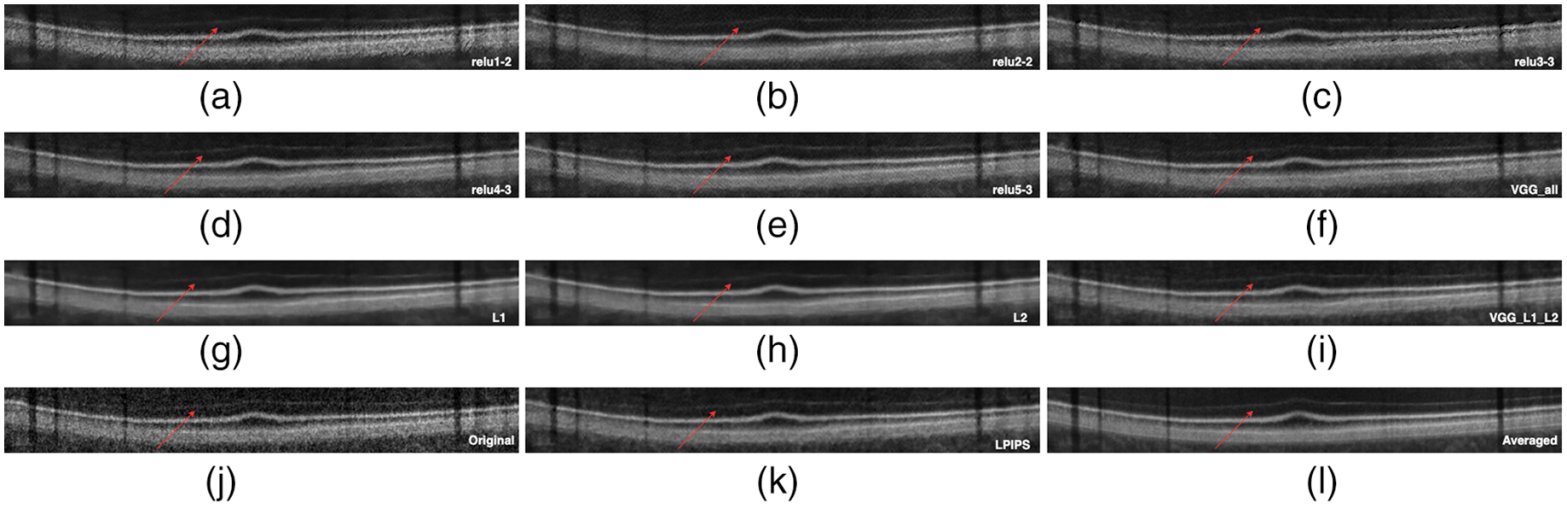
This ROI corresponds to the highlighted red rectangle in Fig. 7. The red arrow points to the ELM retinal boundary. ELM is faded away by the DnCNN-$L_{1}$ and DnCNN-$L_{2}$ networks, but is more visible using VGG feature loss networks.

In this section, we present the visual inspection analysis of the OCT denoised images. Figure 7 shows a visual comparison of the denoised outputs of the trained networks for one OCT B-scan, while Figs. 8–10 show expanded regions-of-interest (ROIs) color coded to those in Fig. 7.

Overall, all networks are capable of reducing the speckle noise in the output images. However, DnCNN-$L_{1}$, DnCNN-$L_{2}$, and DnCNN-VGG-$L_{1}\text{-}L_{2}$ blurred the images more than other networks. This effect can be visually assessed in Figs 8(g), 8(h), and 8(i). It is worth noting the differences between VGG(all) in Fig. 8(f) and $L_{1}/L_{2}$ in Figs. 8(g) and 8(h), where it can be seen that VGG is less blurry but a texture is introduced to the image.

In regards to VGG, examining the output of individual layers may provide more insight into their contribution to performance. The first layer [relu1-2 Fig. 8(a)] appears to be the closest to $L_{1}/L_{2}$ [Figs. 8(g) and 8(h)]. This is perhaps not surprising as it has the least processing effect on the output of the first network, where $L_{1}$ and $L_{2}$ losses are calculated. Layer 2 (relu2-2) appears to have a significant role in introducing texture in VGG [Fig. 8(b)] and subsequent layers [Figs. 8(c)–8(e)]. DnCNN-Conv2-2 introduced artifacts in the form of added textures to the denoised image, while DnCNN-Conv4-3 and DnCNN-Conv5-3 were seemingly preferable because of lack of blurriness, especially compared with DnCNN-$L_{1}$ and DnCNN-$L_{2}$, as can be seen in the zoomed ROIs in Figs. 8 and 9. As for enhancement of faint features, Fig. 10 shows parts of the ILM layer denoised by different networks. DnCNN-$L_{1}$ and DnCNN-$L_{2}$ oversmoothed some fine structures, resulting in loss of meaningful structures such as the ILM layer. On the other hand, as highlighted by the red arrows, the ILM layer is more visible in the VGG-based networks such as DnCNN-Conv4-3 and DnCNN-Conv5-3.

### 3.4 Assessment of OCT Image Sharpness

To quantitatively assess the denoising performance of each network, we quantify OCT image sharpness using two image quality assessment metrics: PSNR and edge preservation index (EPI). In addition, we quantify the denoised OCT images with no-reference objective image sharpness metrics since we do not have access to a “true” reference image. These metrics include the perceptual sharpness index (PSI),^36^ just noticeable blur (JNB),^37^ and spectral and spatial sharpness measure (S3).^38^ These methods report on a single sharpness value, which is representative of sharpness around edges, contrast between layers, and overall perceptual sharpness of the whole image.

#### 3.4.1 Peak signal-to-noise ratio

We employ PSNR as a metric to evaluate the similarity between the denoised OCT image and a reference averaged image. PSNR is defined as follows:

$$\mathrm{PSNR}=10\;\log_{10}(\frac{\max{\left(I\right)}^{2}}{\mathrm{MSE}}),$$ (8)

where max(I) represents the maximum pixel intensity value in image I and MSE is the mean-square-error between the denoised image and the reference image.

#### 3.4.2 Edge preserving index

The EPI, as its name suggests, is intended to reflect the ability of the processing method to preserve the edge details. We calculate the EPI at each retinal layer boundary as

$$\mathrm{EPI}=\underset{i,j}{\sum }|I_{d}(i+1,j)-I_{d}(i,j)|,$$ (9)

where $I_{d}$ represents the normalized pixel value at boundary coordinates (i,j), i represents the i’th row, and j represents the j’th column. The higher EPI values reflect higher accumulative differences between pixel values around retinal boundaries or higher contrast.

It is worth noting that the EPI metric has certain limitations, while assessing the edge sharpness in OCT images. First, EPI is sensitive to noise. Presence of speckle noise makes it hard to interpret the high EPI values as either the result of high contrast around the retinal boundaries or simply a response to fluctuation of intensity around the retinal edges due to noise. Second, EPI simply measures the change in the intensity, while there is no measure of the reference edge position or size.

Because of the orientation on the retinal layer boundaries in the OCT images, we are primarily interested in “horizontal” edges, while generally for layer segmentation there is little boundary information in the vertical direction. The original methods^39^ that used vertical edges were adapted here for the horizontal edges.

### 3.5 Perceptual Sharpness Index

PSI is a blur metric method based on local edge gradients. In the first step of the algorithm, PSI generates an edge map by applying a vertical and horizontal Sobel filter on the image. In the second step, the algorithm estimates the edge widths by pixelwise tracing along the edge gradients. The image is then divided into blocks (e.g., 32×32  pixels) to calculate the (local sharpness estimates) given by

$$\text{local sharpness}=\frac{L}{\omega_{\mathrm{PSI}}},$$ (10)

where $\omega_{\mathrm{PSI}}$ is the measured edge widths within a block and L is the total number of blocks. Then, the global sharpness estimation, the PSI, is calculated as the highest γ’th percentile average of the local sharpness values. In our experiments, γ=22 was used.

### 3.6 Just Noticeable Blur

JNB^37^ is the minimum amount of perceived blurriness around an edge at a specific contrast without being noticed. The higher values of JNB indicate a lower amount of blurriness in a given picture. In the first step, JNB detects the edges using the Sobel operator. The image is then divided into blocks (e.g., 64×64  pixels), where each block is labeled as an edge block if the number of edge pixels is higher than a threshold N (e.g., 2% of the pixels in each block). In step 3, the edge width is calculated. Finally, the perceived blur distortion within an edge block $R_{b}$ is given as

$$D_{R_{b}}={\left(\underset{e_{i}\in R_{b}}{\sum }{\left|\frac{\omega (e_{i})}{\omega_{\mathrm{JNB}}(e_{i})}\right|}^{\beta }\right)}^{\frac{1}{\beta }},$$ (11)

where $\omega_{i}$ is the calculated edge width at pixel “I;” and $3\leq \omega_{\mathrm{JNB}}\leq 5$ is the JNB width, an experimental parameter chosen based on local contrast around the edge |foreground−background|; and β=3.6 is an another experimental hyperparameter.^37^ The overall JNB metric is given as

$$\mathrm{JNB}=(\frac{n}{{\left(\underset{R_{d}}{\sum }{\left|D_{R_{b}}\right|}^{\beta }\right)}^{\frac{1}{\beta }}}),$$ (12)

where n is the total number of blocks in the image.

### 3.7 Spectral and Spatial Sharpness

The S3 measure is a no-reference sharpness measure that yields a local sharpness map in which greater values correspond to greater perceived sharpness within an image and across different images. The $S_{3}$ map can also be represented by a single scalar value that denotes the overall perceived sharpness for a full-sized image by

$$S_{3}=\underset{x\in X}{\max}\;S_{3}(x),$$ (13)

where x is a block in X and $S_{3}(x)$ denotes the sharpness value in the $S_{3}(X)$ map.

The $S_{3}$ sharpness map is calculated based on the combination of spectral-based sharpness map $S_{1}(x)$ and spatial-based sharpness map $S_{2}(x)$ given as

$$S_{3}(X)=S_{1}{\left(X\right)}^{\gamma }\times S_{2}{\left(X\right)}^{1-\gamma },$$ (14)

where 0≤γ≤1 and $S_{1}(x)$ is calculated based on the slope of the magnitude spectrum α of block x by

$$S_{1}(x)=1-\frac{1}{1+e^{-3(\alpha -2)}},$$ (15)

where, given block x, to compute α, first the two-dimensional discrete Fourier transform denoted y(f,θ) is computed, where f is the radial frequency and θ is the orientation. From y(f,θ), next the total magnitude spectrum is computed across all orientations $z(f)=\underset{\theta }{\sum }|y(f,\theta )|$.

The slope of the spectrum of x is then given as

$$\alpha^{*}=\arg\;\underset{\alpha }{\min}{\left\|\beta f^{-\alpha }-z(f)\right\|}_{2},$$ (16)

where $L_{2}$-norm is taken over all radial frequency f.

$S_{2}(x)$ is calculated based on the total variation proposed in Ref. 40. The total variation of block x is given as

$$\nu (x)=\sum_{i,j}|x_{i}-x_{j}|,$$ (17)

where $x_{i}$ and $x_{j}$ are eight-neighborhood pixels in x.

$S_{2}(x)$ denotes a measure of perceived sharpness based on total variation of block v(x) as

$$S_{2}(x)=\underset{\xi \in x}{\max}\;v(\xi ),$$ (18)

where ξ is a 2×2 block of x.

According to Ref. 38, the sigmoid function in $S_{1}(x)$ accounts for the human visual system, where the images with 0≤α≤1 appear sharp and regions with α>1 appear blurred. In the experiments section, we will present our results based on $S_{3}$ and α.

### 3.8 Quantitative Results

To quantify the performance of each network, we calculated the PSNR, PSI, JNB, and S3 for the 20 test images.

The DnCNN-Conv1-2 and DnCNN-Conv5-3 networks produce denoised images with the highest perceptual sharpness metrics. The VGG-based networks outperform the $L_{1}$ and $L_{2}$ networks for all of the image sharpness quality metrics but achieve lower PSNR values. This indicates that $L_{1}$ and $L_{2}$-loss based networks can lower noise levels at the cost of compromising image sharpness, resulting in blurry effects and loss of faint features that are essential for diagnosis. The summary of the metrics is presented in Table 1.

**Table 1:** Quantitative metrics of denoised test images across different networks. For all metrics, higher values indicate superior performance. The bold and italic numbers represent the best and second best performances in each column.

Network	Loss	PSNR	PSI	JNB	S3
DnCNN-$L_{2}$	$L_{2}$	*34.18*	0.214	14.46	0.17
DnCNN-$L_{1}$	$L_{1}$	**34.33**	0.208	14.34	0.16
DnCNN-Conv1-2	VGG layer1	32.43	0.303	**18.07**	**0.41**
DnCNN-Conv2-2	VGG layer2	32.1	0.318	16.91	0.27
DnCNN-Conv3-3	VGG layer3	31.08	0.306	16.5	*0.38*
DnCNN-Conv4-3	VGG layer4	32.09	0.317	17.73	0.30
DnCNN-Conv5-3	VGG layer5	30.63	*0.329*	*17.92*	0.32
DnCNN-VGG	VGG	32.39	0.291	17.47	0.27
DnCNN-$L_{1}\text{-}L_{2}$-VGG	$\lambda_{1}$$L_{1}+\lambda_{2}$$L_{2}+\lambda_{3}$ VGG	33.56	0.237	13.82	0.20
DnCNN-LPIPS	LPIPS	32.36	**0.38**	13.89	0.26
Averaged	—	—	0.27	13.41	0.16

**Fig. 11:**
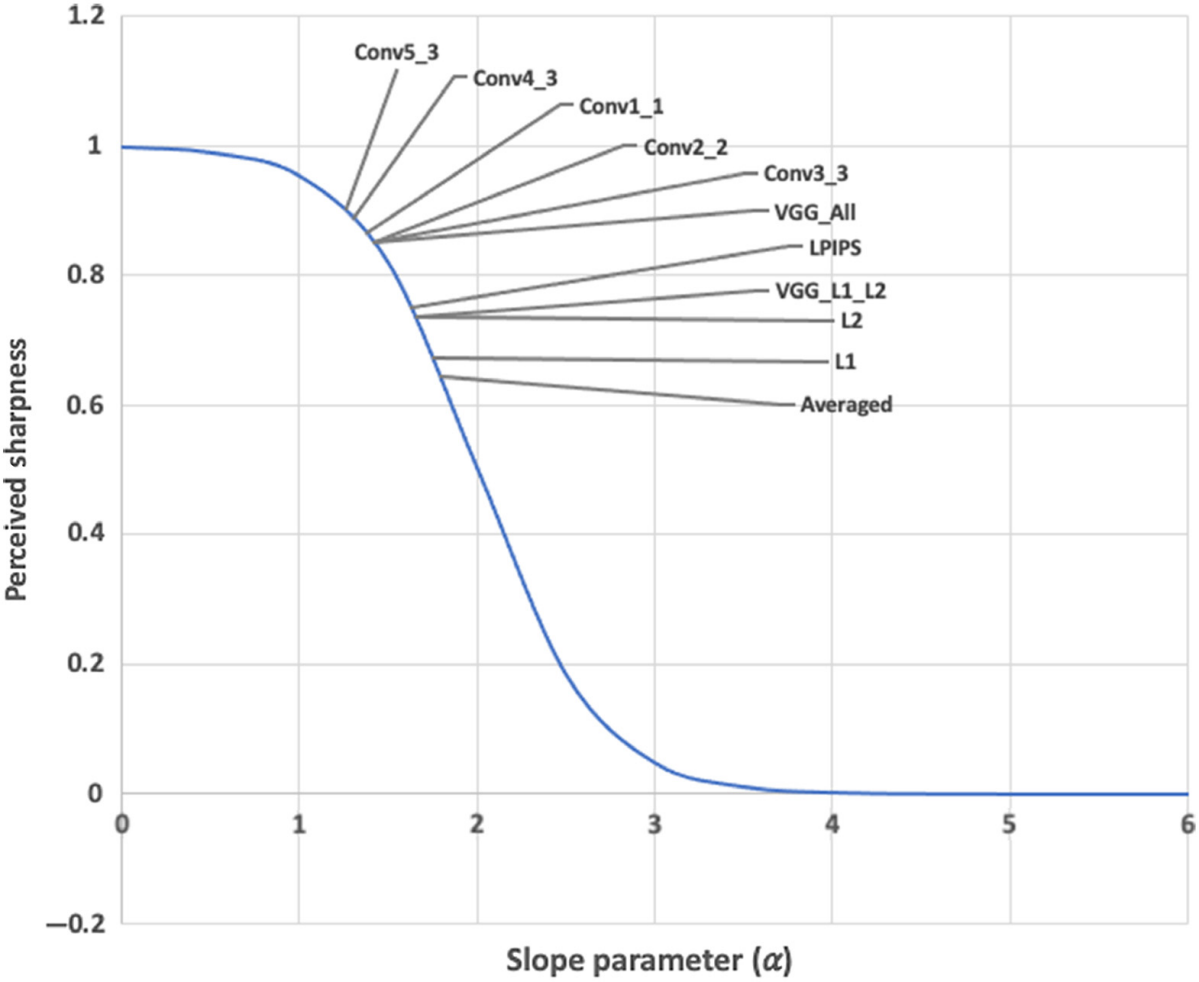
The slope parameter α of each trained network and their relative positions on the perceived sharpness measure spectrum. According to the diagram, the sharpness measure of the images drops rapidly for 1<α<3. An OCT image with 0<α<1 will appear sharp, while OCT images with α>1 will appear progressively blurred as α increases.

Figure 11 shows the perceived sharpness measure of each trained network across the slope parameter α defined in Sec. 3.7. DnCNN-$L_{1}$ and DnCNN-$L_{2}$ have the lowest perceived sharpness measure compared with VGG-based networks, with DnCNN-Conv5-3 exhibiting the highest sharpness measure on the slope parameter spectrum.

**Fig. 12:**
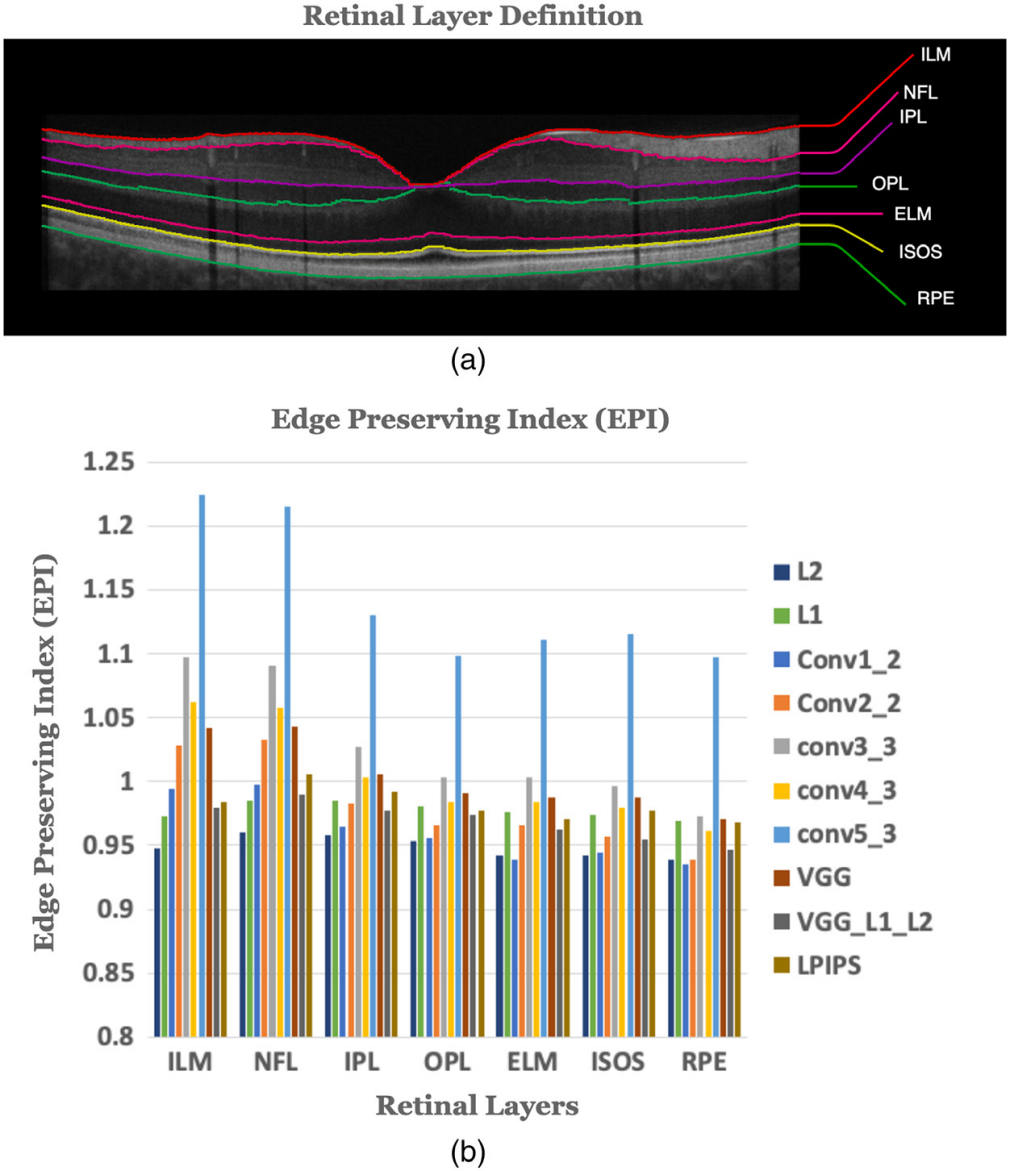
(a) An averaged B-scan OCT image with marked retinal layer boundaries and (b) EPI for test OCT images around retinal layer boundaries.

Figure 12 shows the EPI calculated for the seven retinal layer boundaries. VGG layer 5 consistently exhibits higher (better) EPI compared with other losses over all seven retinal layer boundaries. On the other hand, the pixelwise loss networks score the lowest EPI across all retinal layers. This result is aligned with the no-reference image sharpness measures presented earlier.

## 4 Discussion

We have investigated the effect of deep feature losses of VGG-16 and LPIPS on the denoising performance of the DnCNN network for OCT speckle noise reduction. We focused on the individual layers of the VGG-16 network to distinguish the contribution of each distinctive layer as a loss function, with the aim of producing denoised OCT images that are perceptually clear and preserve the faint features (retinal layer boundaries) essential for diagnosis. Our findings using real clinical images indicate that the perceptual loss can effectively optimize the network to denoise the OCT image by preserving the meaningful anatomical structures of the retina (i.e., layers) and avoiding the blurring and oversmoothing effects produced by networks trained with pixelwise loss functions. All of the denoised images of the networks were reviewed and qualitatively compared against their corresponding averaged OCT image to assess (1) the presence of deep learning induced image blurriness in the denoised images, (2) the presence of deep learning induced textures in the denoised images, (3) overall visibility of retinal layer boundaries, and (4) preservation of fine features that are essential for diagnosis. Networks optimized by $L_{1}$ and $L_{2}$ losses resulted in denoised images with a blurred effect. This visual inspection also revealed that the second layer in VGG (relu2-2) had a significant role in introducing textures in denoised OCT images, while the fourth (relu4-3) and fifth (relu5-3) layers in VGG and LPIPS were better (produced images with crisper retinal boundaries) loss functions compared with $L_{1}$ and $L_{2}$. Similarly, the fine features such as the ILM layer boundary were best preserved with the DnCNN-Conv4-3, DnCNN-Conv5-3, and DnCNN-LPIPS networks. To quantify these findings, we employed a range of metrics (PSNR, EPI, PSI, JNB, and S3) to assess the quality of the denoised OCT images. Consistent with our qualitative analysis, perceptual sharpness measures also showed the highest scores for the DnCNN-LPIPS with the highest PSI (0.38) and DnCNN-Conv1-2 with the highest JNB (18.07) and S3 (0.416). It is worth mentioning that VGG-based loss networks did not achieve high PSNRs similar to $L_{1}$ and $L_{2}$.

This study had some limitations. First, we acknowledge that this study dealt with OCT images obtained from a single OCT instrument. Further training on datasets obtained from different cameras is required to ensure that the network will provide a universal solution. Second, the scope of our experiments was to look into each of the individual layers of the VGG network. Further research is required to assess individual filters for a selective set of deep features suitable for OCT denoising and to reduce any potential unwanted contribution from the layers. Third, expert observers were not included as the scope of our experiments aimed to introduce a nonconventional set of techniques to perceptually quantify the performance of the denoising networks. Further research is required to compare the perceptual sharpness methods with expert observer scores on the OCT denoised images.

## 5 Conclusion

OCT images with high levels of speckle noise can be hard to interpret clinically, so the development of OCT image denoising methods represents a relevant clinical tool. In this work, we have demonstrated that there is a trade-off between smoothness and feature sharpness in selecting the loss function. We demonstrated that indeed some layers (levels of abstraction) of the VGG16 network are more effective than others and more effective than the VGG16 network as a whole for OCT image denoising. Overall, DnCNN-Conv5-3 consistently scored better in sharpness scores and less blurring effect in the resulting denoised OCT images. However, earlier convolutional layers (DnCNN-Conv1-2 and DnCNN-Conv3-3) also exhibited high sharpness scores compared with other networks. As for future work, to further optimize the performance, one might consider a weighting approach that selects a combination of a number of VGG16 layer outputs as a loss function for denoising OCT images. We also experimentally showed that assessing performance only using PSNR may not provide a complete assessment of the method, since feature sharpness is equally important to ensuring that image detail is preserved. The findings of this study highlight the importance of the careful selection of the loss function and bring attention to a new set of evaluation metrics for OCT image denoising.
